# Obesity and inflammation markers in relation to leukocyte telomere length in a cross-sectional study of persons with Barrett’s esophagus

**DOI:** 10.1186/s40608-015-0063-3

**Published:** 2015-09-10

**Authors:** Sheetal Hardikar, Xiaoling Song, Rosa Ana Risques, Thomas J. Montine, Catherine Duggan, Patricia L. Blount, Brian J. Reid, Garnet L. Anderson, Mario Kratz, Emily White, Thomas L. Vaughan

**Affiliations:** Public Health Sciences Division, Fred Hutchinson Cancer Research Center, Seattle, WA USA; Department of Epidemiology, University of Washington, Seattle, WA USA; Department of Pathology, University of Washington, Seattle, WA USA; Human Biology Division, Fred Hutchinson Cancer Research Center, Seattle, WA USA; Department of Medicine, University of Washington, Seattle, WA USA; Department of Genome Sciences, University of Washington, Seattle, WA USA; Department of Biostatistics, University of Washington, Seattle, WA USA; 1100 Fairview Ave. N., M4-B402, PO Box 19024, Seattle, WA 98109-1024 USA

## Abstract

**Background:**

Telomere shortening is associated with increasing age, male gender and lifestyle factors such as obesity and smoking. Inflammation has also been implicated in cellular senescence and may promote telomere shortening in chronic conditions such as obesity and diabetes. However, little is known about the relationship between markers of obesity and inflammation, and leukocyte telomere length (LTL).

**Methods:**

LTL was measured using quantitative polymerase chain reaction in peripheral leukocytes from 295 individuals diagnosed with Barrett’s esophagus (BE) between 1995 and 2009. Data on lifestyle variables including obesity and smoking were collected at in-person interviews. Biomarkers of obesity (leptin, adiponectin), diabetes (glucose, insulin), inflammation (C-reactive protein, Interleukin-6, surface tumor necrosis factor receptor (sTNFR) I & II) and oxidative stress (F2-isoprostanes) were measured in stored blood samples. We examined associations between these covariates and LTL in a cross-sectional analysis using linear and logistic regression models, adjusting for possible confounders.

**Results:**

LTL was significantly associated with age (*r* = −0.30, *p* < 0.001), gender (*r* = 0.14 for females, *p* = 0.01) and inversely associated with cigarette pack-years (*r* = −0.11, *p* = 0.04). Odds of having short LTL were significantly higher for participants in the highest tertile for sTNF-RI (Odds ratio adjusted for age, gender, smoking, and obesity = 2.19; 95 % CI 1.00–4.85, p-trend = 0.02). LTL was not significantly associated with any other lifestyle factors, including smoking or obesity, or other inflammation-, obesity-/diabetes-related biomarkers measured.

**Conclusions:**

Increasing age, male gender, smoking history, and sTNF-RI levels were associated with short LTL among persons with BE but no correlations were observed between LTL and other inflammatory markers or measures of obesity. Larger longitudinal studies are necessary in order to further establish the potential relationships between obesity, inflammation markers and LTL.

## Background

Telomeres are the protective structures that cap the ends of eukaryotic chromosomes, consisting of a large number of tandem DNA repeats bound to a variety of proteins [1]. They protect chromosomes from end-to-end fusion, degradation and atypical recombination [2]. Telomeres shorten with each cell division, and when telomeres reach a critical threshold, cellular senescence is triggered via DNA damage checkpoint mechanisms [1, 2]. Hence, telomere length has been considered as a biomarker for ageing.

Telomere shortening has consistently been associated with older age [3, 4], male gender [3, 4] and Caucasian race [5]. Shorter telomeres have also been shown to be associated with lifestyle factors such as obesity [6], and smoking [6], but these associations have not been consistent across studies [7]. Telomere shortening has been established as a risk factor for chronic conditions such as cardiovascular disease [4] and diabetes [8]. Additionally, telomere shortening has been observed early in precursor lesions for various cancers [9], and epidemiological studies have suggested shortened telomeres as potential biomarkers of cancer incidence and mortality [10, 11], including cancers of the esophagus [12] and stomach [13].

Inflammation and oxidative stress have been associated with ageing in general [14], and shortening of telomeres in particular [15, 16]. Oxidative stress and resulting reactive oxygen species may cause single-stranded breaks in telomeric DNA either directly or indirectly through improper DNA repair [17]. Chronic conditions such as obesity and diabetes may also affect telomere length by promoting inflammation; studies have implicated adipokines in the development of insulin resistance, accelerated cell senescence and ageing [18]. Although it is hypothesized that inflammation may contribute to telomere shortening by accelerating cellular turnover [19] particularly among obese individuals, the relationship between systemic markers of obesity, diabetes, and inflammation, and leukocyte telomere length (LTL) remains to be better elucidated.

Barrett’s esophagus (BE), the only identified precursor of esophageal adenocarcinoma, is a metaplastic condition of the esophagus where the normal squamous epithelium is replaced by a specialized type of columnar intestinal epithelium, usually as a result of chronic gastroesophageal reflux [20]. It is estimated to occur in 2–4 % of adults in the US. Central adiposity has been shown to be a strong and common risk factor for BE; together with gastroesophageal reflux, these risk factors cause chronic inflammation both in the esophageal tissue as well systemically [21].

In this cross-sectional analysis, we evaluated correlations between LTL, measured by quantitative polymerase chain reaction, and demographic and lifestyle factors including obesity and smoking, and systemic biomarkers of obesity, diabetes, and inflammation, in a subset of the Seattle Barrett’s esophagus study (SBES) cohort, who were diagnosed between 1995 and 2009. As most persons with BE are obese and have significant underlying systemic inflammation, this cohort provided a good opportunity to understand the inter-relationships between obesity, inflammation and LTL.

## Methods

### Study population

The SBES is a prospective cohort study aimed at identifying factors that predict neoplastic progression in persons with BE, a precursor to esophageal adenocarcinoma. Participants underwent a personal interview at enrollment along with dietary and anthropometric assessments, endoscopy with biopsies and collection of blood samples, details of which have been previously described [22, 23]. We used baseline data on 295 individuals that had LTL measurements available at baseline, to test cross-sectional associations between LTL and demographic and lifestyle factors. Of the 295 with available LTL measurements, 202 had biomarker measurements available at baseline while 32 had biomarkers measured within a year from their baseline visit. We used biomarker data from these 234 individuals to test the associations between LTL and markers of inflammation, diabetes and obesity. Participants with C-reactive protein (CRP) levels greater than 10 mg/L (*n* = 12) were omitted from analysis involving CRP due to an *a priori* hypothesis that they may be a result of acute inflammation. Institutional Review Boards at the University of Washington and Fred Hutchinson Cancer Research Center approved the study. Written, informed consent was obtained from all the participants prior to study enrollment.

### Covariates

Anthropometric measurements, including body mass index (BMI), waist circumference (WC) and waist-hip ratio (WHR) were measured as described previously [22]. Cigarette use was described as ever use (at least one cigarette/day for six months or longer) and cigarette pack-years of smoking (number of cigarette packs smoked per day times number of years smoked). Alcohol consumption was computed after combining responses for beer, wine and liquor intake. A history of NSAID and statin use was also collected at baseline, as reported previously [23, 24].

### Assays

Participants provided fasting venous blood samples which were processed within 2 hours after collection and stored at −80 °C until analysis. Intra- and inter-assay coefficients of variation (CVs) were calculated by including blind duplicate samples with each laboratory batch, as described previously [25, 26]. Briefly, inflammation markers measured and their intra- and inter-batch CVs are respectively: CRP (immunonephelometry; Dade Behring; inter-batch CV 2.88 %), interleukin-6 (IL-6; Quantikine HS human IL-6 Elisa kit; R&D Systems; CVs 4.13 % and 4.35 %), soluble tumor necrosis factor receptor- I & II (sTNFR-I & II; MILLIPLEX MAP Human Soluble Cytokine Receptor Panel; Millipore; sTNFR-I CVs 5.87 % and 8.93 %, sTNFR-II CVs 2.39 % and 6.09 %), F_2_-isoprostanes (gas chromatography/ mass spectrometry; 6890 N Agilent gas chromatograph & 5973 quadruple mass spectrometer; precision ±3 %). We also measured several obesity- and diabetes-related markers on stored blood samples, details of which have been described previously [27]. These included leptin (Linco Research Human leptin radioimmunoassay; Millipore, Billerica, MA), adiponectin (multimeric enzyme-linked immunosorbent assay; ALPCO Diagnostic, Salem, NH), glucose (Clinical Chemistry Autoanalyzer, using the glucose/hexokinase procedure), and insulin (Tosoh autoanalyzer; Tosoh Bioscience, Inc, San Francisco, CA); the intra- and inter-assay coefficients for these assays ranged from 1.4 to 11.9 %. Homeostatic model assessment (HOMA) score was computed from the insulin and glucose measurements [27]. Laboratory personnel were blinded to other exposures and outcomes.

### LTL analysis

Genomic DNA was extracted from buffy coat preparations stored at −80 °C and telomere length was measured using quantitative polymerase chain reaction [28]. A relative unitless measure of telomere length, T/S ratio, was computed by dividing the amount of telomeric DNA (T) with the amount of single-copy control gene (S). All measurements were performed in triplicate and the median was used for all calculations. In addition, two internal control DNA samples were run within each plate to evaluate inter-plate variation. For T/S ratios, the intra- and inter-assay CVs were 6 % and 7 %, respectively. The mean T/S ratio of the cohort was standardized to have a mean of 0 and a standard deviation of 1.0 to enable comparisons within the cohort.

### Statistical analysis

Medians and standard deviations for continuous variables and proportions for categorical variables were computed by telomere tertiles. Age- and gender-adjusted correlations between various participant characteristics, including biomarker levels, and LTL were investigated with Pearson product–moment correlations as well as linear regression models. LTL was the dependent variable in the linear regression models.LTL and biomarker measures were normally distributed hence were not log transformed but rather used in their original form for analyses. We further assessed whether selected participant characteristics and higher levels of obesity and inflammation biomarkers were associated with increased odds for short telomere length (shortest telomere tertile; T/S ratio < 0.735). Biomarkers were evaluated as continuous measures, and categorized as tertiles. For every variable of interest, three different models were run: unadjusted, age- and gender-adjusted, and further adjusted for smoking and obesity, both major correlates of inflammation [29, 30]. Odds ratios (ORs) and 95 % confidence intervals (CIs) were calculated using unconditional logistic regression models. Tests for trend were based on the likelihood-ratio test associated with addition of the variable of interest in its continuous form. Sensitivity analyses were conducted after dropping those that had biomarker measurements within a year of baseline visit. Results with p-values less than 0.05 were considered to be statistically significant. All statistical analyses were carried out using STATA statistical software (version 12.0; Stata Corp).

## Results

Participant characteristics within telomere tertiles are shown in Table 1 along with results of the correlation and linear regression analysis. LTL was the dependent variable in these analyses. On average, participants were 60.9 years, overweight (average BMI = 29.2 kg/m^2^), predominantly male (80 %), and 66 % were either current or past smokers (66 %).

**Table 1:** Study participant characteristics and their linear associations with leukocyte telomere length^a^

Sample characteristic			Leukocyte telomere length tertile†			Pearson’s correlation coefficient^¶^	Linear regression	
		*n*	Short (*n* = 96)	Middle (*n* = 99)	Long (*n* = 100)		Age & gender adjusted beta (95 % CI)	*P*-value
Leukocyte telomere length (T/S ratio)†		295	0.68 (0.05)	0.78 (0.03)	0.90 (0.08)	-	-	-
Age (per year) †		295	65.5 (10.9)	62.0 (12.0)	57.0 (10.8)	−0.30^₤^	−0.003 (−0.004,-0.002)^₤^	<0.001
Gender ‡	Male	236	84 (87.5)	78 (78.8)	74 (74.0)	-	REF	-
	Female	59	12 (12.5)	21 (21.2)	26 (26.0)	-	0.04 (0.01,0.07)^€^	0.01
BMI (per kg/m^2^) †		290	28.1 (3.6)	29.2 (4.5)	29.1 (4.3)	0.05	0.002 (−0.002,0.005)	0.35
Waist-hip ratio (per unit) †		294	0.95 (0.06)	0.96 (0.07)	0.95 (0.07)	0.05	0.10 (−0.13, 0.34)	0.38
Waist circumference (per inch) †		294	39.2 (4.0)	39.6 (4.9)	39.6 (4.4)	0.09	0.002 (−0.001, 0.005)	0.14
Cigarette use ‡	Never	100	33 (34.4)	29 (29.3)	38 (38.0)	-	REF	-
	Ever	195	63 (65.6)	70 (70.7)	62 (62.0)	-	−0.007 (−0.03, 0.02)	0.64
Cigarette pack-years (per pack-year) †		195	34.0 (25.6)	23.6 (24.8)	18.5 (19.1)	−0.11	−0.0006 (−0.0011,−0.0000)	0.04
Alcoholic drinks/day (per drink) †		240	1.7 (4.9)	1.4 (7.9)	1.0 (2.5)	−0.07	−0.002 (−0.004, 0.001)	0.20
NSAID use ‡	Non-current	190	58 (60.4)	64 (64.7)	68 (68.0)	-	REF	-
	Current	105	38 (39.6)	35 (35.3)	32 (32.0)	-	−0.001 (−0.029, 0.027)	0.93
Statin use ‡	Non-current	265	83 (86.5)	92 (92.9)	90 (90.0)	-	REF	
	Current	30	13 (13.5)	7 (7.1)	10 (10.0)	-	−0.023 (−0.066, 0.020)	0.29

Leukocyte telomere length tertile: Short < 0.735, Middle 0.735-0.846, Long ≥ 0.847

† = Median (Standard deviation), ‡ = Frequency (%), ₤ = Adjusted only for gender, ¶ = Adjusted for age & gender

*BMI* body mass index, *NSAID* non-steroidal anti-inflammatory drugs, *CI* confidence interval

^a^The correlation and regression values are based on leukocyte telomere length as a continuous variable

Gender-adjusted LTL was inversely correlated with age (*r* = −0.30, *p* < 0.0001) with an attrition rate of 0.003 ± 0.001 per year in the T/S ratio. LTL was significantly longer in females compared to males (*r* = 0.14 for females, p =0.01), in an age-adjusted model. There was little evidence of an association between telomere length and any of the obesity-related variables such as BMI (p =0.35), WHR (*p* = 0.38) or WC (*p* = 0.14) in the linear regression models. Cumulative smoking exposure measured as cigarette pack-years smoked negatively correlated with LTL (*r* = −0.11, *p* = 0.04), and the association remained statistically significant after adjustment for age and gender in the linear regression model [β (95 % CI) = −0.0006 (−0.0011,-0.0000); *p* = 0.04]. Use of medications such as NSAIDs and statins, and alcohol consumption were not associated with LTL. Further adjustment for confounding effects of obesity and smoking did not change any results presented in Table 1 (data not shown).

Table 2 displays the correlations and linear associations between biomarkers and LTL. No significant association with LTL was observed in linear regression models with obesity/diabetes-related biomarkers including leptin and adiponectin, glucose, insulin, or HOMA score. While there was a trend towards an inverse correlation between LTL and biomarkers of inflammation, none of these associations were statistically significant. Adjustment by age, gender, smoking and obesity had no effect on the results (data not shown).

**Table 2:** Biomarker concentrations and their linear associations with leukocyte telomere length^c^

Biomarkers		Leukocyte telomere length tertile^b^			Pearson’s correlation coefficient^a^	Linear regression	
	*n*	Short (*n* = 76)	Middle (*n* = 80)	Long (*n* = 78)		Age & gender adjusted beta (95 % CI)	*P*-value
Leptin (ng/ml)	287	7.8 (10.3)	9.5 (12.3)	10.1 (10.3)	−0.01	−0.0001 (−0.002, 0.002)	0.90
Total adiponectin (mcg/ml)	226	5.2 (2.8)	5.4 (2.4)	4.4 (2.5)	−0.01	−0.001 (−0.007, 0.006)	0.88
HMW adiponectin (mcg/ml)	226	1.9 (1.7)	2.0 (1.6)	1.8 (1.6)	0.01	0.001 (−0.010, 0.011)	0.90
Glucose (mg/dL)	226	91.0 (14.6)	91.5 (28.4)	93.0 (30.3)	0.04	0.0002 (−0.0004, 0.0008)	0.52
Insulin (uU/ml)	226	7.2 (5.2)	6.4 (7.7)	6.9 (5.4)	−0.001	−0.0001 (−0.0024, 0.0024)	0.99
HOMA score	226	1.6 (1.3)	1.5 (3.3)	1.6 (1.7)	0.01	0.0003 (−0.006, 0.007)	0.93
C-reactive protein (mg/L)	222	2.1 (2.3)	1.8 (2.5)	2.3 (2.1)	−0.04	−0.002 (−0.009, 0.005)	0.57
Interleukin-6 (pg/ml)	234	2.1 (2.7)	2.0 (2.9)	1.7 (1.6)	−0.10	−0.005 (−0.011, 0.001)	0.11
sTNF-RI (ng/ml)	234	1.5 (0.5)	1.3 (0.4)	1.3 (0.5)	−0.06	−0.016 (−0.050, 0.020)	0.36
sTNF-RII (ng/ml)	234	5.6 (1.6)	5.3 (1.5)	5.0 (1.1)	−0.003	−0.0003 (−0.012, 0.011)	0.96
F2-isoprostanes (pg/ml)	224	54.0 (34.2)	57.5 (26.5)	54.0 (38.3)	−0.04	−0.0002 (−0.0006, 0.0003)	0.53

Leukocyte telomere length tertile: Short < 0.735, Middle 0.735-0.846, Long ≥ 0.847

*sTNF-RI & RII* soluble tumor necrosis factor receptor I & II, *HMW* high molecular weight, *HOMA score* homeostatic model assessment score

^a^Adjusted for age & gender

^b^Median (standard deviation)

^c^The correlation and regression values are based on leukocyte telomere length as a continuous variable

To identify any non-linear associations between short LTL and biomarkers and/or participant characteristics, we examined whether higher levels of biomarkers were associated with increased odds of short LTL in logistic regression models (Table 3). Each one year increase in age increased the odds of short telomeres by 4 % (OR adjusted for gender, smoking and obesity =1.04, 95 % CI 1.02–1.07). Females were 63 % less likely to have short telomeres than males (OR adjusted for age, smoking and obesity =0.37, 95 % CI 0.18–0.78). None of the obesity-related variables (BMI, WHR or WC) or smoking variables (ever use and pack-years) were associated with short LTL in logistic regression models. Analyses on obesity- and diabetes-related biomarkers also did not show any significant results. When viewed on a continuous scale, increasing sTNF-RI levels were associated with increased odds of short LTL (OR adjusted for age, gender, smoking and obesity =2.30, 95 % CI 1.11–4.78. A significant trend was observed with sTNF-RI such that those in the highest tertile were at more than twice increased odds of having short telomeres as compared to those in the lowest tertile (OR adjusted for age, gender, smoking and obesity =2.19, 95 % CI 1.00–4.85, p-trend = 0.02).Although the unadjusted odds for short LTL were significant for sTNF-RII, this association disappeared after adjustment for confounders, including age, gender, smoking, and obesity. There were no significant associations observed between short LTL and higher levels of CRP, IL-6 or F2-isoprostanes.

**Table 3:** Odds ratios (OR) for short LTL^a^ by subject characteristics and markers of inflammation

Characteristic	*N*	Unadjusted OR (95 % CI)	Age & gender adjusted OR (95 % CI)	Age, gender, smoking & obesity^€^ adjusted OR (95 % CI)
Age (per year)	295	1.05 (1.03,1.07)	1.05 (1.03,1.08) ^₤^	1.04 (1.02,1.07) ^₤^
Gender (Females vs. males)	295	0.46 (0.23,0.92)	0.43 (0.21,0.87) ^€^	0.37 (0.18,0.78) ^€^
BMI (per kg/m^2^)	290	0.91 (0.85,0.97)	0.93 (0.87,1.00)	0.96 (0.86,1.07)
Waist circumference (per inch)	294	0.96(0.91,1.02)	0.94(0.88,1.00)	0.94 (0.88,1.00)
Waist-hip ratio (per unit)	294	1.11(0.77,1.60)	0.72(0.45,1.16)	0.92 (0.49,1.74)
Cigarette use (Ever vs. never)	295	1.01 (1.00,1.03)	1.01 (0.99,1.03)	0.99 (0.96,1.03)
Pack-years continuous (per pack-year)	295	1.01 (1.00,1.02)	1.01 (1.00,1.02)	1.01 (1.00,1.02)
Pack-years Tertiles				-
Non-smokers		REF	REF	REF
T1 (0.05-)		0.58 (0.28,1.20)	0.64 (0.30,1.35)	0.64 (0.30,1.37)
T2 (14-)		0.91 (0.47,1.76)	0.79 (0.40,1.57)	0.84 (0.42,1.70)
T3 (36-)		1.58 (0.83,3.01)	1.30 (0.66,2.56)	1.40 (0.70,2.77)
p-trend^b^		0.04**	0.27	0.20
Leptin (ng/ml)	226	0.98 (0.95,1.01)	1.00 (0.97,1.04)	1.05 (1.01,1.12)
Total Adiponectin (mcg/ml)	226	1.08 (0.97,1.20)	1.00 (0.88,1.15)	0.98 (0.85,1.12)
HMW Adiponectin (mcg/ml)	226	1.10 (0.92,1.30)	0.98 (0.79,1.21)	0.92 (0.74,1.15)
Glucose (mg/dL)	226	0.99 (0.98,1.01)	0.99 (0.97,1.01)	0.99 (0.97,1.01)
Insulin (uU/ml)	226	1.00 (0.95,1.04)	1.00 (0.95,1.05)	1.02 (0.97,1.07)
HOMA score	226	0.95 (0.82,1.09)	0.95 (0.81,1.12)	1.01 (0.86,1.18)
CRP continuous (mg/L)	222	0.98 (0.87,1.11)	0.95 (0.82,1.09)	0.98 (0.84,1.13)
CRP Tertiles				
T1 (0.1-)		REF	REF	REF
T2 (1.1-)		1.36 (0.67,2.74)	1.40 (0.66,2.96)	1.69 (0.78,3.68)
T3 (2.9-)		0.91 (0.45,1.85)	0.81 (0.37,1.78)	1.04 (0.45,2.40)
p-trend^b^		0.74	0.44	0.74
IL-6 continuous (pg/ml)	234	1.06 (0.95,1.17)	1.02 (0.90,1.15)	1.04 (0.92,1.18)
IL-6 Tertiles				
T1 (0.4-)		REF	REF	REF
T2 (1.5-)		1.45 (0.74,2.86)	1.21 (0.58,2.50)	1.25 (0.59,2.62)
T3 (2.6-)		1.25 (0.63-2.47)	1.07 (0.50,2.31)	1.22 (0.55,2.71)
p-trend^b^		0.32	0.77	0.51
sTNF-RI continuous (ng/ml)	234	3.30 (1.75,6.23)	2.02 (1.02,3.99)	2.30 (1.11,4.78)
sTNF-RI Tertiles				
T1 (0.3-)		REF	REF	REF
T2 (1.2-)		1.14 (0.56,2.33)	0.90 (0.42,1.91)	1.00 (0.46,2.15)
T3 (1.5-)		3.25 (1.64,6.43)	1.81 (0.85,3.84)	2.19 (1.00,4.85)
p-trend^b^		<0.001**	0.04**	0.02 **
sTNF-RII continuous (ng/ml)	234	1.30 (1.07,1.58)	1.08 (0.86,1.35)	1.06 (0.84,1.33)
sTNF-RII Tertiles				
T1 (2.1-)		REF	REF	REF
T2 (4.9-)		0.90 (0.44,1.82)	0.64 (0.30,1.37)	0.68 (0.32,1.47)
T3 (6.0-)		2.52 (1.29,4.90)	1.42 (0.66,3.04)	1.45 (0.67,3.15)
p-trend^b^		0.01**	0.50	0.62
F2-isoprostanes continuous (pg/ml)	224	1.00 (0.99,1.01)	1.00 (1.00,1.01)	1.01 (1.00,1.02)
F2-isoprostane Tertiles				
T1 (14-)		REF	REF	REF
T2 (48-)		0.62 (0.31,1.25)	0.67 (0.32,1.41)	0.72 (0.34,1.52)
T3 (67-)		0.93 (0.48,1.83)	1.28 (0.60,2.71)	1.55 (0.71,3.39)
p-trend^b^		0.88	0.31	0.18

*LTL* leukocyte telomere length, *BMI* body mass index, *CRP* c-reactive protein, *IL-6* interleukin-6, *sTNF-RI & RII* Soluble tumor necrosis factor receptor I & II, *CI* confidence interval

£ - Adjusted for cigarette pack-years smoked at baseline, € - Adjusted for waist circumference at baseline, ₤ = Adjusted only for gender, € = Adjusted only for age

** p-value significant at 0.05 level

^a^Short LTL defined as lowest tertile (T/S ratio < 0.735)

^b^Test for trend based on the likelihood-ratio test associated with addition of the variable under consideration in its continuous form

To better understand the interrelationship between obesity and inflammation markers in relation to LTL, we also evaluated the effect of adjustment for obesity on the relationship between inflammation markers and telomere length as well as the effect of adjustment for inflammation markers on the obesity-telomere length relationship for all the models. We observed that further adjustment for obesity increased the odds for short telomeres (e.g. further adjustment for obesity, as measured by waist circumference, increases the OR for shortest sTNF-RI telomere tertile from 2.00 (95 % CI 0.99–4.03) to 2.30 (95 % CI 1.11–4.78) after adjusting for obesity.

We also conducted separate sensitivity analyses after excluding measurements from 32 individuals that had their biomarkers measured within one year of baseline so as to completely exclude the possibility of reverse causality. We found that the linear association for cigarette pack-years of smoking got slightly weaker (*r* = −0.06) and did not hold statistical significance after age and gender adjustment. The positive result for sTNF-RI also did not change significantly when we excluded the 32 individuals whose measurements were taken within one year of baseline (Adjusted OR = 2.32, 95 % CI 0.98–5.46). The trend for sTNF-RI tertiles also remained significant even after dropping the 32 measurements (p-trend = 0.04).

## Discussion

In this cross-sectional study we found evidence linking a serum-based marker of inflammation, elevated sTNF-RI, with shortened LTL. Individuals with sTNF-RI levels in the highest tertile were at 2.2 times increased odds of having short LTL compared to those individuals with sTNF-RI levels in the lowest tertile. We also found suggestive evidence of a trend of shorter LTL with increasing sTNF-RII levels, although this was not significant after adjustment for confounders. As we expected, we also observed associations between LTL and age, gender and smoking.

Our findings are in line with previous research regarding associations of LTL with demographic and lifestyle characteristics. A recent review by Sanders *et. al.* summarized the utility of telomere length as a biomarker for ageing in epidemiological studies and established increasing age and male gender to be most consistently associated with shorter LTL across studies [7]. The evidence regarding smoking has been mixed [7]. Some studies have reported a positive association with LTL [31], while others have reported negative association [3, 4].

To date, there are limited data on the association between inflammatory biomarkers and LTL. Reports that have observed an association between increasing levels of inflammation markers and short LTL are few in number [4, 15, 16, 32]. One hypothesis that may explain a cross-sectional association between LTL and inflammation suggests that inflammation may accelerate telomere attrition by enhancing leukocyte turnover and replicative senescence [33]. Shorter telomeres may also cause programmed cell death and lead to accumulation of excessive senescent cells which in turn may be responsible for the elevated levels of inflammation markers [19, 34]. A factor supporting this hypothesis is that the main sources for TNF-α secretion (and thereby shedding of the soluble TNF receptors), fibroblasts and mononuclear cells, are both involved in clearing of the senescent cells from the body [35]. In the present analysis, we only found associations with LTL for sTNF receptors, particularly sTNF-RI. Moreover, this association was apparent only in logistic regression models (no association was found in linear models). We are not sure of the robustness of this finding with sTNF-RI as the cut-points for both short telomeres and sTNF receptor tertiles were arbitrary, and this significant finding with sTNF-RI may be entirely data driven. Additionally, we also evaluated interrelationships between obesity and inflammation with respect to LTL. We observed that the age, gender, and smoking adjusted OR’s became stronger after further adjustment for obesity in inflammation-LTL association models. These results suggest that visceral adiposity and inflammation markers may independently influence telomere length. However, as these results are based on a cross-sectional analysis, any inferences on temporality should be drawn with caution, and further evaluation in a longitudinal setting is necessary to better understand this relationship.

Very few studies have evaluated the association of LTL with obesity/diabetes-related biomarkers such as leptin, adiponectin [36, 37], or insulin, glucose or HOMA scores [8, 38]. The rationale behind these studies is similar to that behind studies of the inflammation-LTL relationship i.e. the presence of these chronic conditions may give rise to increased turnover of cells, and ultimately shorter telomeres. Results from previous studies show positive associations with some obesity and diabetes markers and negative associations with others, that do not seem to be patterned on populations studied [7]. A recent meta-analysis reported an inverse association with leptin and no association with adiponectin [37]. However, this study only adjusted for confounding effects of BMI and was not able to adjust for central adiposity measures such as waist-hip circumference or waist circumference. In addition to adjusting for a likely more predictive measure of adiposity (waist circumference), we were also able to adjust for smoking, an important confounder in the association between obesity and LTL. Our results did not show a significant association of LTL with any of the obesity/diabetes-related biomarkers evaluated. We were not able to replicate the inverse association with leptin demonstrated in the meta-analysis by Broer et. al [37]. This may be due to a smaller sample size of our study but may also be due to a more complete adjustment for confounding in our study relative to the meta-analysis.

Our study was limited by the cross-sectional design as it does not allow for temporal interpretations. The sample size is also limited. This may partially contribute to why we were unable to observe any association between telomere length and obesity/diabetes. There is also a possibility that the association observed with sTNF-RI might be a chance finding as we conducted multiple statistical tests in the limited sample size available. The study participants were a high-risk cohort of BE patients, many being obese (38 %). This might have resulted in higher levels of both obesity and inflammation markers among the study participants as compared to the general population [30]. This can be viewed upon as both a strength and a limitation. On the one hand, we did not encounter the problem of a large proportion of biomarkers being below detection limit, while on the other hand, the higher inflammation levels in this cohort make the results from this study less generalizable to other populations. Although we controlled for confounding in multivariable analysis, there is a possibility of residual confounding. In addition, we cannot exclude the possibility of measurement error in the estimation of biomarkers, driving the risk estimates towards the null (assuming non-differential measurement error). Strengths of our study include a well characterized study population with detailed information on important confounders and reliable laboratory measurements judged by blinded QC samples embedded in the study samples.

## Conclusions

In summary, the present study shows that the inflammation marker sTNF-RI, is significantly associated with short LTL among persons with BE. In addition, we confirmed previous findings of shortened telomeres with increasing age, male gender and increasing pack-years of smoking. No association was observed between biomarkers of obesity/diabetes and short LTL. In order to further establish the potential relationship between obesity, inflammation and telomere length, larger longitudinal studies are needed.
